# Assessing characteristics of RNA amplification methods for single cell RNA sequencing

**DOI:** 10.1186/s12864-016-3300-3

**Published:** 2016-11-24

**Authors:** Hannah R. Dueck, Rizi Ai, Adrian Camarena, Bo Ding, Reymundo Dominguez, Oleg V. Evgrafov, Jian-Bing Fan, Stephen A. Fisher, Jennifer S. Herstein, Tae Kyung Kim, Jae Mun (Hugo) Kim, Ming-Yi Lin, Rui Liu, William J. Mack, Sean McGroty, Joseph D. Nguyen, Neeraj Salathia, Jamie Shallcross, Tade Souaiaia, Jennifer M. Spaethling, Christopher P. Walker, Jinhui Wang, Kai Wang, Wei Wang, Andre Wildberg, Lina Zheng, Robert H. Chow, James Eberwine, James A. Knowles, Kun Zhang, Junhyong Kim

**Affiliations:** 1 Department of Genomics and Computational Biology, Perelman School of Medicine, University of Pennsylvania, Philadelphia, PA USA; 2 Department of Chemistry and Biochemistry, University of California at San Diego, La Jolla, CA USA; 3 Department of Psychiatry & The Behavioral Sciences, Keck School of Medicine, University of Southern California, Los Angeles, CA USA; 4 Department of Physiology & Biophysics, Zilkha Neurogenetic Institute, University of Southern California, Los Angeles, CA USA; 5 Illumina, Inc., San Diego, CA USA; 6 Department of Biology, University of Pennsylvania, 415 S. University Ave, Philadelphia, PA 19104 USA; 7 Department of Pharmacology, Perelman School of Medicine, University of Pennsylvania, Philadelphia, PA USA; 8 Present address: Allen Institute for Brain Science, Seattle, WA USA; 9 Department of Bioengineering, University of California at San Diego, La Jolla, CA USA; 10 Department of Neurological Surgery, Zilkha Neurogenetic Institute, University of Southern California, Los Angeles, CA USA

**Keywords:** Single-cell RNA-sequencing, Biotechnology, Bioinformatics, Genomics

## Abstract

**Background:**

Recently, measurement of RNA at single cell resolution has yielded surprising insights. Methods for single-cell RNA sequencing (scRNA-seq) have received considerable attention, but the broad reliability of single cell methods and the factors governing their performance are still poorly known.

**Results:**

Here, we conducted a large-scale control experiment to assess the transfer function of three scRNA-seq methods and factors modulating the function. All three methods detected greater than 70% of the expected number of genes and had a 50% probability of detecting genes with abundance greater than 2 to 4 molecules. Despite the small number of molecules, sequencing depth significantly affected gene detection. While biases in detection and quantification were qualitatively similar across methods, the degree of bias differed, consistent with differences in molecular protocol. Measurement reliability increased with expression level for all methods and we conservatively estimate measurements to be quantitative at an expression level greater than ~5–10 molecules.

**Conclusions:**

Based on these extensive control studies, we propose that RNA-seq of single cells has come of age, yielding quantitative biological information.

**Electronic supplementary material:**

The online version of this article (doi:10.1186/s12864-016-3300-3) contains supplementary material, which is available to authorized users.

## Background

Single-cell RNA sequencing (scRNA-seq) allows unprecedented resolution for studies of gene expression. Since its introduction in 2009 [1], this approach has been used to identify and classify cell types, characterize rare cells, and study expression variation across cell populations [2–10]. In this method, the RNA content of a single cell is captured, reverse transcribed to generate cDNA, amplified and sequenced, providing measurements of the transcriptomes of single cells with nucleotide-level resolution. Compared with methods to sequence bulk RNA, scRNA-seq requires substantial molecular amplification and consequently, additional handling and enzymatic reactions. This has the potential to introduce additional experimental errors and molecular biases, such that analytic methods designed for bulk RNA sequencing may not be appropriate for single-cell measurements. Despite substantial experimental methods development [11–16], the effects of RNA amplification methods remain complex and poorly characterized. Though measurement characteristics likely depend on the specific experimental protocol used, there has been limited dissection of factors that affect input–output relationship across methods. (Although see notable exceptions [17–20], considered further in *Discussion*.)

Here, we first describe our model-based approach to dissect the factors that affect the amplification step of scRNA-seq and then we characterize expression measurements generated by three scRNA-seq methods in terms of sensitivity, precision and accuracy. We find that all methods perform comparably overall, but that individual methods demonstrate unique strengths and biases.

## Results

### Method overview

Our approach was to dilute bulk total RNA (from a single source) to levels bracketing single-cell levels of total RNA (10 pg and 100 pg) in replicates and amplifying the RNA to levels sufficient for RNA sequencing. Here, we analyzed the performance of scRNA-seq methods in terms of sensitivity (number of unique gene models detected), precision (replicate variation), and accuracy (deviation from bulk). We note that expected replicate precision depends on the exact sequence of dilutions that lead to the final set of replicates. For example, if a single “master” dilution mix is made from which *n* replicate final dilutions are created, the expected number of molecules for each replicate will be based on the master dilution, not the original bulk. Each replicate value in relation to the bulk will be comprised of two terms, the variance term due to the final dilutions and a bias term, which is the deviation of the master dilution from the bulk. Different experimental protocols (e.g., using Fluidigm C1 to generate replicates) require attention to the expected variation. We employed a general linear model framework to dissect the factors governing measurement performance. In particular, we observed that certain genes, or even control ERCC probes, have a tendency for large deviations from expectations and we created a list of problematic gene models for future reference. We add the caveat that our results are not reliable outside the range of experimental values from which we fitted the models and the inferences should be interpreted with care.

### RNA-sequencing datasets

**Fig. 1:**
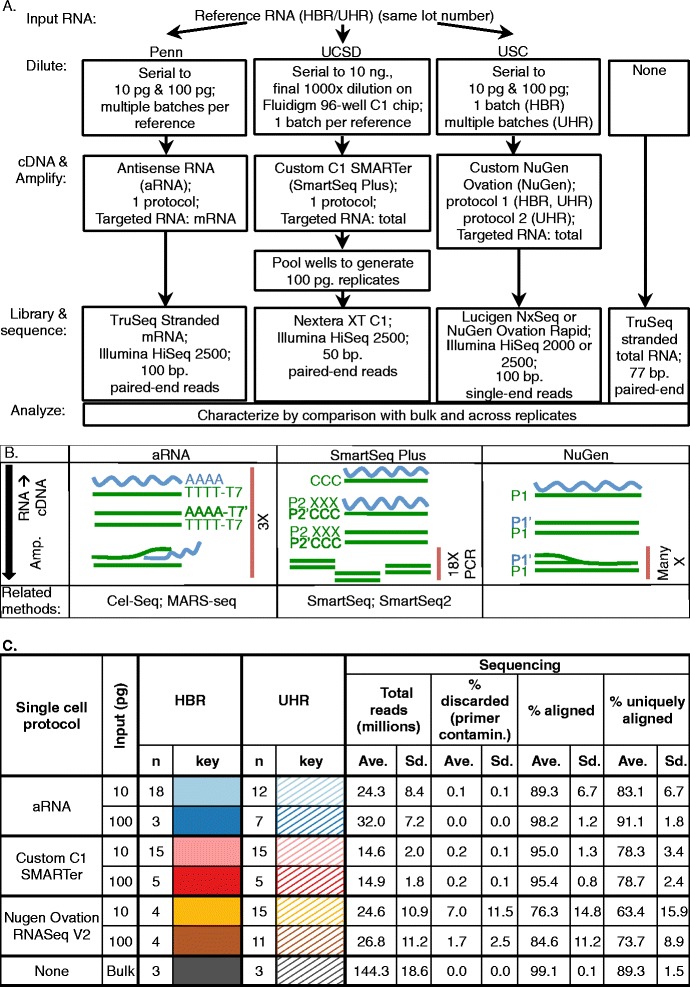
Experimental design and RNA sequencing statistics by experimental group. **a** Dilution experiment summary. See *Methods* for detailed information. **b** Single cell amplification methods used. Protocols involve two key steps: conversion of RNA (blue) to cDNA (green), and amplification of cDNA. aRNA targeted poly-adenylated mRNA by using an oligo-dT T7 primer for initial cDNA synthesis. After generating double-stranded cDNA, molecules were amplified using in vitro transcription with T7 polymerase. This amplification procedure was designed to minimize exponential expansion of errors. cDNA generation and amplification were repeated two additional times before library preparation. SmartSeq Plus targeted total RNA using a mixture of poly-T and randomized primers for initial cDNA synthesis. Full-length transcripts were captured through the template-switching capacity of reverse transcriptase. Double stranded cDNA molecules were amplified using 18 rounds of PCR. All cDNA and amplification reactions were performed on a 96-well Fluidigm C1 chip, intended to reduce experimental variation by performing reactions in small volume. NuGen targeted total RNA through use of proprietary primers for initial cDNA synthesis. Second strand cDNA synthesis was generated using an RNA primer, which was subsequently degraded from the second strand cDNA copy, resulting in linear amplification by DNA replication. This method was designed to minimize exponential amplification of error. **c** Sample sizes and RNA sequencing statistics by experimental group. Includes color key used in all figures. For analysis based on combined HBR and UHR dilution replicates, solid colors were used. Abbreviations: Human Brain Reference (HBR), Universal Human Reference (UHR), University of Pennsylvania (Penn), University of California San Diego (UCSD), University of Southern California (USC), picogram (pg.), base pair (bp.), contamination (contamin.), average (Ave.), standard deviation (Sd.), amplification (amp.)

We performed replicate transcriptome amplifications of Universal Human Reference RNA (UHR) and Human Brain Reference RNA (HBR) that were diluted to single-cell and ten-cell abundances (10 and 100 picograms (pg.) total RNA or ~200,000 and 2 million mRNA molecules, respectively) and were amplified using three single-cell RNA amplification methods (Fig. 1a–b). Methods included the antisense RNA IVT protocol (aRNA), a custom C1 SMARTer protocol (SmartSeq Plus) performed on a Fluidigm C1 96-well chip, and a modified NuGen Ovation RNA sequencing protocol (NuGen, Fig. 1b-c, Additional file 1). Bulk ribo-depleted UHR and HBR RNA were sequenced and served as a reference. The general experimental scheme was consistent for all dilution replicates; however, there were differences across experimental groups in the specifics of experimental protocols, necessitated by particular methodologies (Fig. 1a, see *Methods* and Additional file 1 for full details). Because of these experimental differences, head-to-head comparison of methods is not appropriate and our goal is to provide quantitative analyses of factors affecting individual methods. Current results should be used in experimental planning, data analysis, and method optimization rather than as a performance test of any particular method.

### Data processing

Briefly, all samples were aligned to the human reference genome (hg19) using STAR aligner [21] and splice-site annotations from GENCODE18. Uniquely aligned reads were assigned to GENCODE18 gene annotations using HTSeq and htseq-counts [22] and then were depth normalized [23]. Ribosomal genes and genes with short isoforms (<300 nucleotides) were excluded because of differences in sequencing protocols across groups (Fig. 1a), leaving 42,855 genes for analysis. (We use “gene” to match GENCODE18 gene ids, a set that includes both coding and non-coding RNA). To avoid artifacts caused by alignment or quantification ambiguities, we generated a stringently filtered gene list containing 10,039 genes to which reads can be uniquely assigned and referred to these genes as “computationally unambiguous” throughout (Additional file 2). Reference RNA were aligned and quantified with RSEM (RNA-seq by Expectation-Maximization) [24]. Estimated abundances were concordant with publicly available PrimePCR measurements and with poly-A RNA sequencing measurements (Additional file 3, SEQC/MAQC-III Consortium, 2014, GEO accession numbers: GPL18522, GSM1362002-GSM1362029, GSM1361974-GSM1362001 [25]).

For each gene, we calculated the expected number of input molecules in a diluted replicate. We did this in three steps (detailed in *Methods*). Briefly, first we estimated the mass of targeted input RNA in diluted replicates as in Brennecke et al. [2]. Second, we estimated the total number of molecules in a diluted replicate using the mass of targeted input RNA and the average transcript mass for each HBR and UHR transcriptome (calculated based on the average transcript length weighted by expression level). Third, we found the expected number of molecules for each gene in a diluted replicate by multiplying the relative frequencies of gene expression and the total number of molecules in a diluted replicate. For each HBR and UHR, transcript lengths and relative expression levels were estimated by RSEM using bulk sequencing data. This approach relies predominantly on bulk sequencing data for estimation, with the exception of estimating the mass of targeted RNA in a dilution replicate. Because of this, we expect these estimates to be relatively robust to unknown biases in scRNA-seq measurements. In particular, these estimates do not depend on the choice of length normalization method for scRNA-seq data. aRNA selectively targeted poly-adenylated (poly-A) mRNA (Fig. 1a). We calculated the expected number of input poly-A molecules using publicly available bulk HBR sequencing measurements.

On average, replicates were sequenced at a depth of 22.0 ± 9.6 million reads (± standard deviation or Sd.). 1.5 ± 5.3% of reads were discarded due to primer contamination. 89.3 ± 10.6% of retained reads aligned to the genome, 77.6 ± 11.2% uniquely (Fig. 1c). To examine the coverage distribution of each method, we quantified the frequency of mapped reads over several genomic regions of interest (Table 1). This distribution differed for the three single-cell amplification methods. The majority of aligned reads for aRNA dilution replicates originated from non-mitochondrial exons (excluding rRNA), a substantially larger proportion than that recovered by SmartSeq Plus or NuGen. rRNA genes, pseudogenes and repeats encoded by the nuclear genome comprised a small fraction of reads in all amplified libraries (average ± SD: 0.67 ± 0.65%). rRNA and mRNA encoded by the mitochondrial genome (2 genes and 13 genes, respectively) constituted a substantial percentage of reads (average ± SD: 16.5 ± 8.4%). Mitochondrial recovery differed substantially across methods. This difference may translate into a method-specific effect on depth normalization and for this reason mitochondrial genes have been excluded from the subsequent analyses. The distribution of reads across genomic features also differed substantially across replicates for aRNA and NuGen (Table 1).

**Table 1:** Coverage selectivity by method

Source	Protocol	Genome coverage									
		% exons (excluding rRNA & mitochondria)		% intronic		% rRNA (nuclear)		% rRNA (mitochondrial)		% mitochondrial (non-rRNA)	
		Ave.	Sd.	Ave.	Sd.	Ave.	Sd.	Ave.	Sd.	Ave.	Sd.
HBR	aRNA	59.07	5.70	23.29	4.68	0.03	0.02	4.82	1.62	12.29	2.79
	SmartSeq Plus	41.00	0.86	39.56	0.85	1.28	0.07	10.02	0.43	7.77	0.26
	NuGen	29.27	4.97	45.09	5.88	1.33	0.40	20.38	5.19	3.65	0.59
	Bulk (Poly-A)	80.13	-	8.52	-	0.08	-	1.96	-	9.01	-
	Bulk (rRNA-depleted)	61.44	0.54	37.89	0.45	0.03	0.01	0.10	0.04	0.25	0.04
UHR	aRNA	71.57	2.34	23.02	2.60	0.06	0.05	1.61	0.19	3.38	0.59
	SmartSeq Plus	35.89	0.89	52.84	0.94	0.31	0.02	6.20	0.26	3.85	0.12
	NuGen	33.00	4.02	39.33	8.99	1.25	0.57	23.47	7.31	2.59	0.62
	Bulk (Poly-A)	86.99	-	7.11	-	0.11	-	0.47	-	5.09	-
	Bulk (rRNA-depleted)	58.17	0.34	41.28	0.29	0.02	0.01	0.03	0.01	0.18	0.02

Average percent of aligned reads assigned to genomic regions for each method. Nuclear rRNA includes rRNA genes, pseudogenes and repeats. See *Methods* for definitions of genomic regions

### Gene detection sensitivity

**Fig. 2:**
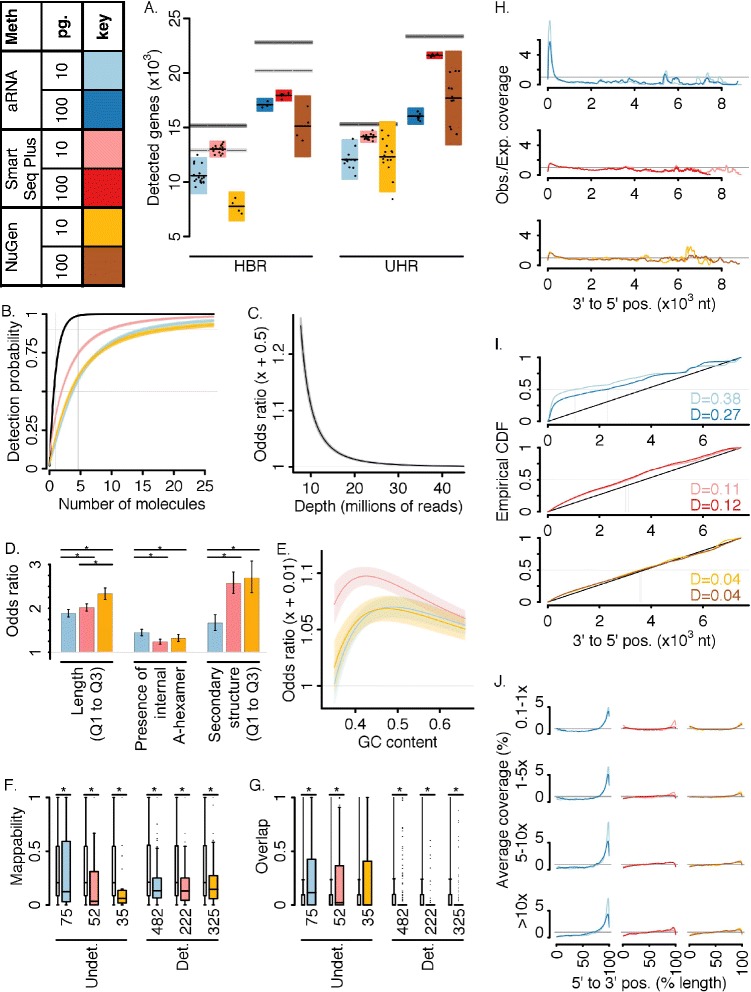
Single-cell RNA sequencing sensitivity. **a** Number of detected genes. Each point represents a single sample. Horizontal black lines indicate group mean. Boxes indicate ± 2 Sd.. Gray horizontal lines indicate 95% CI for the expected number of genes in a diluted replicate, assuming total (*dark gray*) or poly-A (*light gray*) RNA. See *Methods*. **b** Probability of gene detection as a function of the expected number of input molecules estimated using logistic model (see main text and *Methods*). Horizontal lines indicate 50% and 90% probability. Vertical lines indicate 1 and at 4.605 molecules (99% probability of ≥ 1 molecule present in diluted replicate). Bands indicate 95% CI. Black line indicates probability of ≥ 1 molecule present in a diluted replicate. **c** Odds ratio for gene detection as a function of sequencing depth (+500,000 reads). Horizontal line indicates an odds ratio of one (no gain in detection sensitivity). Band indicates 95% CI. **d** Odds ratios for differences in biophysical trait values. Error bars indicate a 95% CI. “*” indicates significant difference across pairs of methods (Bonferonni corrected *p* < 0.05). The odds ratio for length and secondary structure are shown for the increase from 25%ile length (structure) in the HBR and UHR transcriptomes to 75%ile length (structure). **e** Odds ratio for an increase of 0.01 in GC content. Bands indicate 95% CI. **f** Boxplot of gene mappability, or the fraction of the gene body that can be aligned to uniquely (see *Methods*) for computationally ambiguous gene detection outliers (*wide* boxes) and background genes (*narrow* boxes). Both undetected (Undet.) and detected (Det.) outliers are shown. “*” indicates significant difference (Wilcoxon rank-sum two-way test *p* < 0.05). **g** As in F, but for the fraction of the gene body that overlaps in genomic position with a separate gene annotation. **h** Nucleotide coverage. Observed over expected coverage normalized for expression level as a function of absolute 3' to 5' position. See *Methods*. **i** Comparison of nucleotide coverage with uniform distribution. Empirical CDF is of normalized per nucleotide coverage. Black diagonal line indicates uniformity. Kolmogorov-Smirnov (KS) statistic for difference from the uniform distribution is in the bottom right, with larger values indicating greater difference between the distributions. **j** Coverage for genes with different expression levels. Relative 5' to 3' coverage, calculated over 100 equally spaced bins for four expression level categories (rows). See *Methods*. *Abbreviations:* confidence interval (*CI*); Cumulative distribution function (*CDF*)

We calculated the number of detected genes as a measure of detection sensitivity (Fig. 2a) and compared this statistic to the number of genes expected to be present initially in a diluted replicate (horizontal lines in Fig. 2a). While ~30,000 genes were expressed in each bulk HBR and UHR sample (TPM > 0), a diluted replicate should contain many fewer genes because of the sampling that occurs during dilution. For this reason, detection sensitivity should be assessed with respect to the number of genes expected to be present in the diluted replicate, rather than the number of genes observed in the bulk (as is common practice). Briefly, to calculate the expected number of genes in a diluted replicate, we assumed that the number of molecules in a tube for a given gene is Poisson distributed with mean equal to the expected number of molecules for that gene in a diluted replicate, and that genes are sampled independently during dilution. The presence or absence of a given gene in a diluted replicate follows a Bernoulli distribution, with the probability of success equal to the probability that at least one molecule for the gene is in the diluted replicate. The number of genes in a diluted replicate is then drawn from a Poisson-Binomial distribution. See *Methods* for further details.

All methods demonstrate comparable high gene detection, detecting greater than 70% of the number of genes expected to be present in a diluted replicate. SmartSeq Plus demonstrated the highest detection (Byar’s 95% C.I., Obs./Exp.: aRNA (0.722, 0.726); SmartSeq Plus (0.877, 0.882); NuGen (0.735, 0.740)). With respect to poly-A RNA, aRNA detected (0.840, 0.844) of expectation. Variation across samples within each method was substantially larger than expected due to dilution suggesting additional loss during cDNA and amplification (Fig. 2a). To see whether gene detection sensitivity depended on sequencing depth, we generated random low-depth *in silico* samples by randomly sampling 500,000 genic read counts for each dilution replicate. This represents approximately a 20-fold lower sequencing depth and a comparable depth to many recent studies. Significantly fewer genes were detected at this lower sequencing depth (Additional file 4; aRNA (0.457, 0.460); SmartSeq Plus (0.642, 0.646); NuGen (0.517, 0.520)). We note that this lower sensitivity is due to a difference in sequencing depth only, not the single-cell RNA amplification methods.

Detection of a given gene may depend on parameters such as the input number of molecules, GC-content, presence of internal adenosine monophosphate (A) hexamers, length, strength of molecular secondary structure, and sequencing depth. To estimate the contribution of these factors to gene detection, we fit a logistic regression model to the 10 pg. gene detection data with gene detection as the dependent variable, considering only computationally unambiguous genes to focus on experimental sensitivity. (See *Methods* and Additional files 5 and 6 for details.) All methods had a 50% probability of gene detection at ~2–4 expected input molecules, controlling for the remaining covariates (Fig. 2b, Additional file 7). We calculated a molecular recovery rate as the predicted probability that a gene with 1 expected input molecule will be detected, scaled by the probability that at least one molecule of such a gene will be in a diluted replicate. Molecular recovery rates were greater than 0.25 for all methods (95% prediction interval: aRNA (0.262, 0.279), SmartSeq Plus (0.534, 0.558), NuGen (0.315, 0.339)). With respect to poly-A RNA, aRNA recovery rate was (0.320, 0.349).

Despite the small number of total (targeted) RNA molecules in a single 10 pg. dilution replicate (estimated here to be ~300,000 molecules), sequencing depth had a highly significant effect on gene detection (Additional file 5). Fig. 2c shows the odds ratio of increasing sequencing depth by 500,000 reads. An odds ratio significantly greater than 1 indicates an increase in the odds of gene detection with increasing depth. The odds of gene detection increased substantially with sequencing depth until a depth of ~15–20 million reads or ~50 reads per input molecule. As sequencing depth increases, the odds ratio approaches one, indicating progression towards saturation in gene detection with increasing depth. Here, increasing sequencing depth from 10 to 15 million reads translated into an expected gain of 25.02% in detected genes. The influence of remaining covariates on gene detection differed across methods (Fig. 2d–e). The odds of gene detection increased with gene length for all methods (Fig. 2d). The odds of detecting a gene with a length of 2.40 kilobases (75%ile length across reference transcriptomes) were at least 1.8 times greater than the odds of detecting a 0.681 kilobase gene (25%ile length) for all methods. NuGen demonstrated a significantly stronger length effect than aRNA or SmartSeq Plus (Fig. 2d). For NuGen, the odds of detecting a gene with 75%ile length were 2.33 times greater than the odds of detecting a gene with 25%ile length. The presence of an internal A-hexamer positively influenced the probability of gene detection for all methods, with strongest effect for aRNA. For aRNA, the odds of detecting a gene containing an internal A-hexamer was 1.45 times greater than the odds of detecting a gene without one. Decreased strength of secondary structure increased the odds of detection for all methods, with significantly smaller effect for aRNA than for SmartSeq Plus or NuGen. For aRNA, the odds of detecting a gene with a secondary structure strength of −20.8 kcal/mole (75%ile strength) was 1.67 times greater than the odds of detecting a gene with a strength of −29.1 kcal/mole (25%ile strength). For NuGen and SmartSeq Plus, the odds of detecting a gene with 75%ile strength was at least 2.6 times the odds of detecting a gene with 25%ile strength. While GC content influenced detection probability in a complex manner, SmartSeq Plus demonstrated the strongest GC effect (Fig. 2e).

A small fraction of computationally unambiguous genes had poor fit by the logistic model (0.30 ± 0.14%; see Additional file 8 for a list of outliers and *Methods* for details). Each outlier was categorized as “detected” if the gene was unexpectedly observed and “undetected” if it was unexpectedly missing. Nearly all identified outliers (16/17) were method-specific. A larger proportion of computationally ambiguous genes were poorly fit by the model (3.21 ± 0.23%, Additional file 8) with a sizable fraction (19.81 ± 2.90%) that fit poorly for all methods. These outlier genes had significantly lower fraction of the gene body that could be aligned uniquely than background genes (Fig. 2f; Wilcoxon rank sum two-way test, *p* < 0.05). This was the case for both detected and undetected outliers, indicating that alignment ambiguities likely generate both false positives and false negatives. Outliers also significantly differed from background in the fraction of the gene body that overlaps with another gene annotation, with lower overlap among detected outliers and greater overlap among undetected outliers (Fig. 2g).

To characterize read coverage at the scale of individual base positions, we calculated the observed/expected nucleotide coverage as a function of absolute 3′ to 5′ basepair position within a gene (Fig. 2h). Briefly, to examine the effect of absolute position on coverage, genes were aligned from the 3′ end. For each gene, per nucleotide coverage was normalized such that a uniform distribution of reads along a gene would be assigned a value of one at all positions (see *Methods*). Figure 2h–i are plotted from 3′ to 5′ to reflect the alignment of transcripts by their 3′ end due to common 3′ anchoring in amplified mRNA. Coverage for all methods was significantly different from uniform (Fig. 2i; Kolmogorov-Smirnov test *p* < 10^−10^ for all groups); however, NuGen demonstrated the greatest uniformity (Fig. 2h–i) with similar positional coverage distribution for 10 pg. and 100 pg. dilution replicates. aRNA preferentially covered the 3′ terminal and demonstrated greater 3′ bias for 10 pg. dilution data. SmartSeq Plus showed an intermediate degree of bias. Segregated by expression levels, we found preferential recovery of the 5′ and the 3′ gene ends for low abundance genes and preferential 3′ coverage for high abundance genes (Fig. 2j).

### Precision

**Fig. 3:**
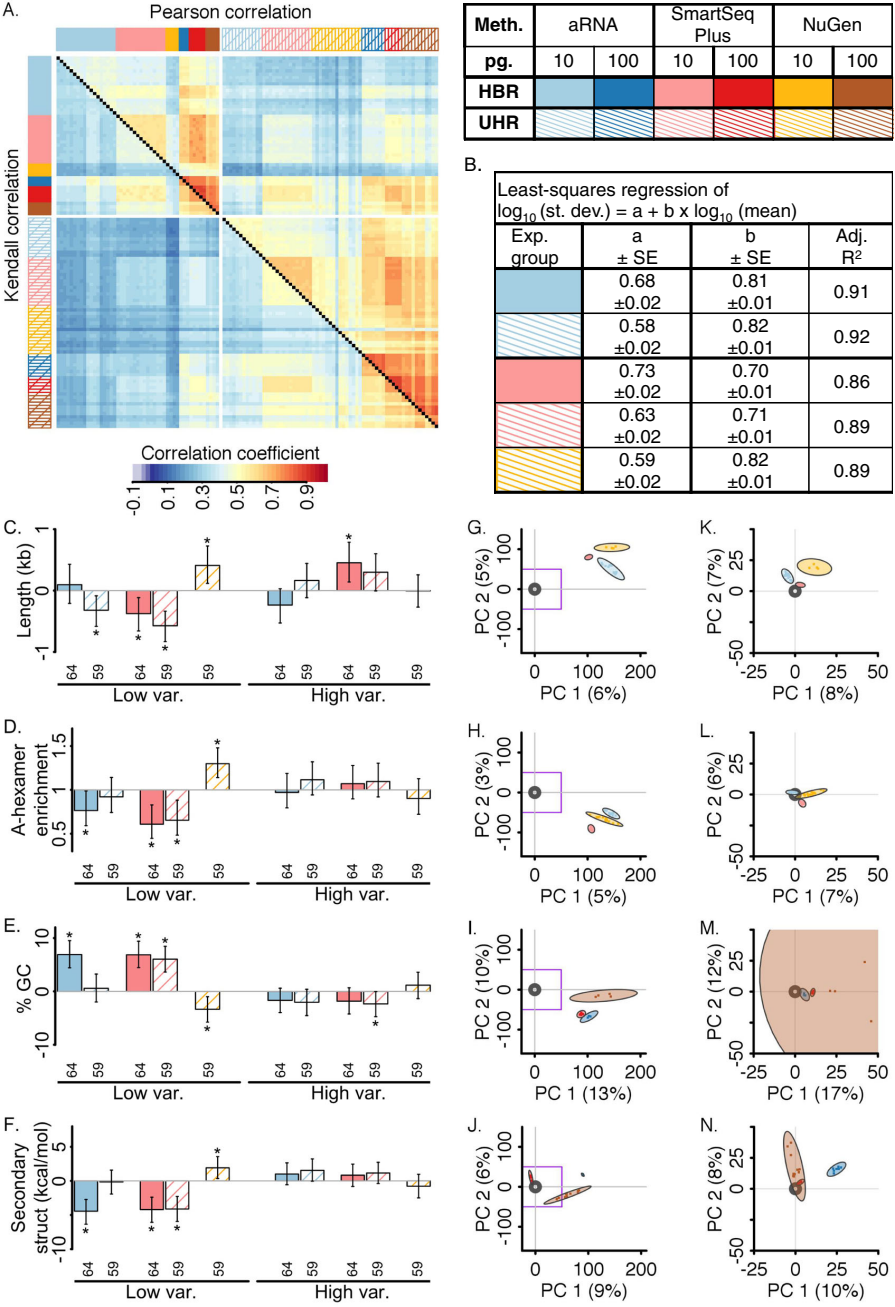
Single-cell RNA-sequencing precision. **a** Pairwise correlations for all samples. Upper triangle: Pearson correlation. Lower triangle: Kendall correlation. Zeros treated as missing values. Each row and column is an individual sample. Experimental group is indicated by color bars at edge of plot. **b** Relationship between standard deviation (*st.* dev.) and mean characterized by least squares regression (see *Methods*). All estimated coefficients were highly significant (coefficient *t*-test *p* < 10^−16^). **c**–**f** Enrichment of biophysical traits in experimentally precise (*low*) and variable (*high*) genes with respect to background genes (see *Methods*). Error bars indicate 95% CI. “*” indicates significant difference (*p* < 0.05). Numbers at bottom indicate sample size (number of genes). (C) Median difference in gene length estimated by Hodges-Lehman statistic. Significance: Wilcoxon rank sum two-way test. (D) Relative risk of containing an internal A-hexamer. Significance: Fisher’s exact test. (E) As C for % GC content. (F) As C for strength of local secondary structure. **g**–**j** PCA projection of dilution data on PC 1 and 2. Plots were centered so that bulk UHR or HBR was positioned at the origin. Points represent individual dilution replicates. Colored ovals represent bivariate normal 95% confidence ellipses. % Sd. explained by a PC is indicated in axis label. See *Methods*. (G) HBR 10 pg. (H) UHR 10 pg. (I) HBR 100 pg. (J) UHR 100 pg. **k**–**n** As G–J, but using only abundantly expressed genes (see main text). Axis scales differ from G–J, with axes in equivalent to the purple-boxed region in G–J. (K) HBR 10 pg. (L) UHR 10 pg. (M) HBR 100 pg. (N) UHR 100 pg. *Abbreviations*: Standard error (*SE*); confidence interval (*CI*); kilobases (*kb*); kilocalories (*kcal*); mole (*mol*); principal components analysis (*PCA*); principal component (*PC*); standard deviation (*sd*)

We next consider the similarity of measurements across dilution replicates, within methods and across methods. The correlation measurements indicate the degree of linear relationship (Pearson coefficient) or consistent ordering (Kendall’s coefficient) over replicate pairs. Though we avoid direct comparison across methods (see Fig. 1a), the results will be applicable to experimental design and analysis for each method. For example, these values may serve as benchmarks for methods optimization, or they may serve as a reference point for new adopters of these protocols to ensure the protocol is being performed adequately. The average within experimental group pairwise correlation coefficient (± Sd.) was 0.37 ± 0.07 (Kendall) and 0.51 ± 0.09 (Pearson, log_10_ counts) for 10 pg. replicates and 0.64 ± 0.06 (Kendall) and 0.79 ± 0.06 (Pearson, log_10_ counts) for 100 pg. replicates (Fig. 3a; zeros treated as missing values).

To describe the dependence of precision on expression level, we performed least-squares regression of the empirical standard deviation on the empirical mean (both variables log-transformed because of their approximate multiplicative scale) for 10 pg. experimental groups with sample size >5. The mean was an excellent predictor of standard deviation (Fig. 3b, adjusted R^2^ > 0.85 and slope coefficient *t*-test *p* <10^−16^ in all cases). To test whether there was systematic bias in variability, we classified a subset of genes to be less precise than expected (top 5% residual values) and another subset to be more precise than expected (bottom 5% residual values). Genes that were less precise than expected differed little from background in their biophysical characteristics (Fig. 3c–f), suggesting limited systematic bias in experimental variability. Biophysical characteristics enriched among unexpectedly precise genes with respect to background differed in a method-specific manner (Fig. 3c–f). For aRNA and SmartSeq Plus, enriched biophysical characteristics were concordant with reduced probability of gene detection (compare Fig. 2d-e), suggesting technical dropouts might play a strong role in replicate precision. NuGen demonstrated the opposite trend suggesting that amplification bias might play a stronger role. Genes with highly atypical precision (top or bottom 1% residual values) are listed in Additional file 9. We recommend that the expression values of these gene models should be interpreted with caution.

Separate principal components analysis (PCA) of each HBR and UHR for 10 pg. dilution data demonstrated that average displacement between single cell and bulk measurements predominate over differences between single cell methods (Fig. 3g–h); however, there were clear differences across methods in the multivariate covariance structure of experimental variation. Differences across methods were also apparent for 100 pg. dilution replicates (Fig. 3i–j), and, though these measurements were more similar to bulk measurements, differences between dilution replicates and bulk measurements persisted. We note that average displacement between single cell and bulk measurements represent both a bias component from utilizing a master dilution mix (see above) and technical bias. We repeated PCA on a subset of genes with greater than 18.5 expected input molecules (expected probability of detection for “typical” gene > 0.9 for all methods). On highly abundant genes, dilution replicates were substantially more similar to bulk measurements (Fig. 3k–n) and differences across methods were substantially smaller. However, in all cases, the within method pattern of covariation (direction of ellipses) and the bias dispersal around the bulk expected value (position of the centroid of the ellipses) differed for both source RNA and individual methods. (We note that bivariate normal 95% confidence ellipse for NuGen 100 pg. replicates is substantially larger than the others (brown oval in Fig. 3m). These samples demonstrate larger spread than matched samples in other experimental groups; however, because the number of replicates is small (*n* = 4) some of this difference may be attributed to sampling noise.)

### Accuracy

**Fig. 4:**
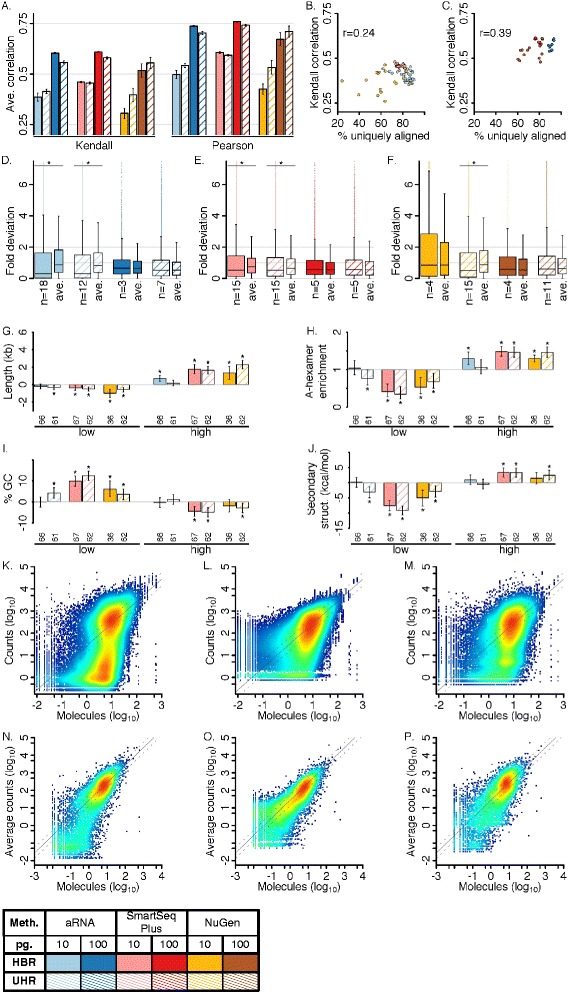
Single-cell RNA sequencing accuracy. **a** Average pairwise correlation of diluted replicates with bulk HBR or UHR. Zeros treated as missing values. Error bars indicate ± 2 s.e.m. **b**–**c** Relationship between % unique alignment and similarity with bulk HBR or UHR. “r” indicates Pearson correlation of x and y. (B) 10 pg. (C) 100 pg. **d**–**f** Distribution of fold deviation across genes. *Wide* boxes represent measurements in individual replicates. *Narrow* boxes represent average measurements across replicates. Y-axis was truncated for visualization and 99%ile values for wide boxes are in panel descriptions below, ordered to match plot. “*” indicates significant difference between individual and average measurements (Wilcoxon rank sum test of greater fold deviation in average measurements, *p* < 0.05). (D) aRNA. 99%ile values: 2066; 1442; 514; 718. (E) SmartSeq Plus. 99%ile values: 877; 770; 553; 598. (F) NuGen. 99%ile values: 2332; 1766; 784; 937. **g**–**j** Enrichment of biophysical traits among underestimated (*low*) and overestimated (*high*) genes with respect to remaining genes. See *Methods*. Plot notation and statistics are as in Fig. 3c–f. (G) Median difference in gene length. (H) Relative risk of containing an internal A-hexamer. (I) As G, for % GC content. (F) As G, for strength of local secondary structure. **k**–**m** Density scatter plots of normalized read counts in individual 10 pg. replicates vs. expected number of input molecules. See *Methods*. Red indicates high density. Solid line indicates expected read count and hashed lines indicate ± 2 fold. (K) aRNA. (L) SmartSeq Plus. (M) NuGen. **n**–**p** Density scatter plots of average normalized read counts vs. number of input molecules. (N) aRNA. (O) SmartSeq Plus. (P) NuGen. *Gene filtering*: D–F and K–P considered computationally unambiguous genes and excluded gene detection outliers. D–J considered genes with greater than 95% probability of presence in a diluted replicate. *Abbreviations*: confidence interval (*CI*); kilobases (*kb*); kilocalorie (*kcal*); mole (*mol*)

We calculated pairwise correlation coefficients of dilution replicates with bulk as a metric of overall accuracy (Fig. 4a). For this and the below, only non-zero gene counts were considered in order to focus on quantitation rather than sensitivity. 10 pg. dilution replicates demonstrated an average pairwise correlation with reference of 0.42 ± 0.01 (Kendall) and 0.55 ± 0.01 (Pearson, log_10_ counts). 100 pg. replicates showed greater similarity with reference (0.57 ± 0.01 (Kendall) and 0.72 ± 0.01 (Pearson, log_10_ counts). Methods demonstrated similar overall accuracy for 100 pg. dilution replicates. For 10 pg. dilution replicates, SmartSeq Plus demonstrated slightly higher correlation with the bulk than the other methods by both correlation metrics. Correlation with reference had a modest association with percent unique alignment (Fig. 4b–c).

To assess the accuracy of individual gene estimates, we calculated the fold deviation of normalized read counts with respect to bulk HBR or UHR measurements (Fig. 4d–f, *Methods*). For all methods and input amounts, the median fold deviation was less than 1 but a subset of genes was extensively overestimated. Overestimated genes (top 5% fold-deviation) were substantially longer than remaining genes and more frequently contained an internal A-hexamer (Fig. 4g–j). For NuGen and SmartSeq Plus, these genes also had lower GC content and weaker local secondary structure than remaining genes. Underestimated genes (bottom 5% fold-deviation) demonstrated the opposite tendencies: compared to background genes, they were shorter, less frequently contained internal A-hexamers, had higher GC content and stronger secondary structure than background, as might be expected (Fig. 4g–j). Overall, aRNA demonstrated less systematic bias than NuGen or SmartSeq Plus. Highly inaccurate genes (top or bottom 1% fold-deviation) are catalogued in Additional file 10.

Smoothed density scatter plots demonstrated method-specific transfer functions between the expected number of input molecules and the number of read counts in an individual replicate (Fig. 4k–m). This relationship was quantitative at expression levels greater than ~5–10 expected input molecules up to at least ~600 input molecules, the highest expression level examined for 10 pg. replicates, giving a quantitative dynamic range of at least 100-fold. This relationship was qualitatively similar for low-depth *in silico* samples (Additional file 11). At this range of expression levels, the relationship between the average number of read counts and the expected number of input molecules is roughly linear (Fig. 4n–p). At low to mid expression levels measurements were frequently underestimated expanding the apparent range of measured abundances, particularly for aRNA and NuGen (Fig. 4n–p).

### Protocol variations

We evaluated the effects of several protocol variations on measurement quality (Table 2). The aRNA protocol used for the primary analysis includes cDNA purification before initial amplification, and 3 rounds of IVT amplification followed by dilution of amplified cDNA before library preparation (Fig. 1b). Elimination of initial cDNA purification significantly improved sensitivity and accuracy, as did reduction to two rounds of IVT amplification and elimination of dilution prior to library generation (Table 2). An optimized protocol incorporating both changes, demonstrated substantial improvements in the number of detected genes and pairwise correlation with the bulk (Table 2).

**Table 2:** Evaluation of protocol variations

Variation	Category	Trait	Modified group		Control group		Median difference (Modified: Control)			Test		
			Sample size	Median	Sample size	Median	Statistic	95% C.I.		Paired/Unpaired	Statistic	*p*-value
No initial cDNA purification	Sensitivity	# genes detected	6	11072	6	10249	782.5	293.0	1678.0	Unpaired	36	0.002
	Precision	Pairwise correlation across samples	15	0.344	15	0.306	0.039	0.028	0.051	Unpaired	215	1.79E-06
	Accuracy	Pairwise correlation with reference	6	0.400	6	0.383	0.019	0.008	0.049	Unpaired	36	0.002
Reduce rounds of cDNA amp.	Sensitivity	# genes detected	5	11936	3	11062	1051.0	33.0	4122.0	Unpaired	15	0.036
	Precision	Pairwise correlation across samples	10	0.329	3	0.333	−0.006	−0.034	0.018	Unpaired	12	0.692
	Accuracy	Pairwise correlation with reference	5	0.430	3	0.401	0.029	0.009	0.066	Unpaired	15	0.036
Optimized aRNA	Sensitivity	# genes detected	5	11936	18	10286	1810.0	942.0	3354.0	Unpaired	84	0.002
	Precision	Pairwise correlation across samples	10	0.329	153	0.306	0.019	0.002	0.035	Unpaired	1084	0.028
	Accuracy	Pairwise correlation with reference	5	0.430	18	0.377	0.055	0.028	0.083	Unpaired	79	0.009
Add ERCCs	Sensitivity	# genes detected	5	10154	4	10101	138.5	−397.0	798.0	Unpaired	13	0.556
	Precision	Pairwise correlation across samples	10	0.287	6	0.282	0.002	−0.012	0.013	Unpaired	34	0.713
	Accuracy	Pairwise correlation with reference	5	0.364	4	0.369	−0.003	−0.016	0.019	Unpaired	9	0.905
Perform strand-specific sequencing	Sensitivity	# genes detected	17	10006	17	10325	−267.0	−331.5	−218.5	Paired	0	1.53E-05
	Sensitivity	Depth of unique genes	17	6.397	17	0.848	5.46	3.68	6.75	Paired	153	1.53E-05
	Sensitivity	# genes not in bulk	17	534	17	610	−62.0	−76.0	−49.5	Paired	0	1.53E-05
	Accuracy	Pairwise correlation with reference	17	0.362	17	0.376	−0.013	−0.017	−0.009	Paired	2	4.58E-05

Comparison of dilution replicates generated using modified protocols with control dilution replicates. Sample information can be found in Additional file 1 and protocol information in *Methods*. # genes detected only considers genes observed in bulk HBR or UHR. Kendall correlation was calculated excluding zeros in either sample. Unpaired comparisons were made using Wilcoxon two-way rank sum test for difference in medians. Paired comparisons were made using Wilcoxon two-way rank sign test for difference in medians. The null hypothesis of no difference was rejected at *p* < 0.05. Median difference between groups, with 95% CI, was calculated using the Hodges-Lehman statistic

The addition of ERCC spike-in transcripts provides an internal control [26], but it raises the concern that addition of synthetic RNA to a sample may decrease biological sensitivity. We found no significant difference in sensitivity, precision or accuracy across matched dilution replicates with and without the addition of ERCCs (Table 2) up to the spike-in level of 2.7% of reads. Individual ERCC transcripts were found to be problematic, consistently inaccurate, for SmartSeq Plus and aRNA in a method specific manner (Additional file 12).

Strand-specific RNA sequencing may improve detection sensitivity and reduce false positive detection. Stranded quantification of aRNA replicates detected slightly fewer genes than non-stranded quantification; however, it also detected significantly fewer genes that were not observed in the bulk, and genes that were detected only by stranded quantification were supported by significantly more reads than genes detected only by non-stranded quantification (Table 2).

## Discussion

Several other publications have reported on similar dilution experiments to assess scRNA-seq methods, where bulk RNA was diluted to small input amounts and amplified in replicate, and the sequencing results of amplified replicates were compared to sequencing results of the bulk material [17, 18, 20]. In comparison to these earlier studies, our study uses a smaller amount of input RNA (compare 10 pg. with 50–500 pg. input total RNA) and a larger number of technical replicates (compare 12 10 pg. UHR replicates with 1 to 3 technical replicates previously) as well as a set of parametric models to dissect the factors affecting statistical characteristics of amplified RNA measurements. Despite these differences, some results presented here are consistent with earlier findings. Of particular interest, both Adiconis et al. [17] and Shanker at al. [20] reported large differences between NuGen and Smart-Seq measurements in the proportion of reads aligning to rRNA, with a large proportion of NuGen reads aligning to mitochondrial rRNA. Because of the larger number of replicates used here, we were able to further observe large variation across replicates generated by the same method in the proportion of reads assigned to mitochondrial rRNA (Table 1).

In contrast with previous studies, we evaluated method performance in terms of the number of input molecules and considered the variance introduced by dilution. When these factors are ignored, it may appear that methods perform more poorly for lower input amounts but this is not necessarily the case. For example, fewer genes were detected in 10 pg. dilution replicates compared to 100 pg. replicates (Fig. 2a). Ignoring the effect of dilution, one may conclude that detection sensitivity is lower for smaller input amounts of RNA; however, because of dilution, fewer genes are expected to be present in 10 pg. dilution replicates than 100 pg. replicates. With this in mind, we cannot conclude that detection sensitivity is lower for smaller input amounts of RNA. More generally, measurement sensitivity (and to some extent precision and accuracy) depends critically on the method’s efficiency of capturing individual molecules (Fig. 2b), which requires careful calibration to match different input level treatments.

Our study, like those described in [17, 18, 20], provides an assessment of scRNA-seq methods with respect to the RNA amplification component of the protocol (including reverse transcription efficiency under ideal conditions). Additional experimental factors will be present in biological applications that are expected to affect the performance of these methods, such as variability across biological samples in the efficiency of cell lysis or an effect of cell debris on cDNA capture, amplification or sequencing. Biological characteristics, such as the amount of lipid contained in a cell, may also affect the efficiency of molecular reactions and introduce further technical variability in resulting measurements. Further study using carefully controlled populations of cells of a variety of cell types as input would extend the analysis performed here.

In light of the results presented here, we briefly discuss a few topics related to experimental planning, method optimization and data analysis.

Though the goal of this study and our experimental design is not meant to select “the best method,” some results may be helpful in selecting an appropriate method for a particular project. The enriched coverage of exons in aRNA may be beneficial for studies of mRNA, and the retention of transcript strand information is unique to aRNA at this point. SmartSeq Plus and C1 microfluidic device generate reproducible replicates and high detection sensitivity, presumably due to more uniform liquid handling and retention of material due to lack of vessel transfer. The uniformity of coverage provided by NuGen (Fig. 2h–i) may be beneficial for studies of isoform use and splicing. We note that, in our hands, NuGen reactions were inconsistent and we had repeated amplification failures, or amplification of non-template directed products with this method, especially at the 10 pg level where the method appears to be reaching the limits of its sensitivity.

In selecting sequencing depth, there is a trade-off between gene detection sensitivity and cost. Typically, a small number of genes comprise the bulk of RNA molecules in a transcriptome. Sequencing at low depths should be sufficient to reproducibly detect and quantify these abundant genes; however, the majority of genes in a typical transcriptome are at low abundance. Because of this, the number of genes detected in the mixtures of RNAs used here depends heavily on sequencing depth (Fig. 2c and Additional file 4). The dynamic range limit due to sequencing depth is a function of the relative frequency distribution, which will vary for an actual single cell. Our results suggest that increasing the number of reads per cell may produce richer transcriptome measurements and should be considered carefully in the context of a specific experimental plan.

Missing values due to lack of sensitivity and the presence of large valued outliers may cause complications for depth normalization methods. Large variation across samples and substantial differences across methods in the fraction of reads assigned to mitochondrial RNA (Table 1) will propagate to sample and method differences in relative read counts. More generally, we observed large variation in the distribution of reads across broad genome annotation classes (Table 1). Because each genomic annotation class accounted for a substantial number of reads and input molecules, the observed differences across methods, and within methods, cannot be simply explained by sampling error. Similarly, variation across samples in the number of detected genes cannot be easily explained by dilution (Fig. 2a). This behavior might be explained by global differences in reaction efficiencies across samples, as suggested previously [27]; however, the experimental sources of such differences in a controlled experiment are unclear. We found certain subsets of genes to be problematic for gene detection, accuracy, and precision, in a method-specific manner (Additional files 8, 9 and 10). We recommend that genes on these lists be treated with caution, filtered before analysis, or interpreted with care. We similarly found several ERCC spike-in transcripts to be problematic (Additional file 12), and recommend selecting a subset of reliable ERCC transcripts for use as reference measurements.

Some scRNA-seq quantification challenges might be reduced through further experimental optimizations, for example by increasing detection sensitivity and reducing amplification biases. Eliminating the initial cDNA purification, reducing the extent of amplification required, and limiting sample dilution may be productive avenues, as suggested by our data. Methods to experimentally deplete highly abundant and variably recovered mitochondrial RNA, if not of experimental interest, may also be of use.

## Conclusions

Single cell RNA measurement methods have become increasingly robust and automated systems have made the technique broadly more accessible and efficient. All methods examined here demonstrated good gene detection and a quantitative relationship between input molecular abundances and measured expression levels at mid- to high-expression levels or greater than ~5–10 input molecules. This corresponds to ~4,000–8,000 reliably measured genes for the reference transcriptomes examined here. We propose that single cell RNA measurements have come of age and this level of resolution for gene expression measurements has and will continue to facilitate biological discovery.

## Methods

### Experimental design

Each collaborating center obtained reference RNA with the same lot number for Universal Human Reference (UHR) RNA (Agilent 740000, Lot 0006141415) and Human Brain Reference (HBR) (Ambion AM6050, Lot-105P055201A) and performed replicate amplification using a single amplification method, detailed below.

#### SmartSeq Plus

Reference RNA was diluted to an intermediate stock solution by serial dilution. A final 1000-fold dilution occurred on the C1 chip, such that individual wells in a given batch contained 9.99 pg. sampled from a common intermediate dilution. ERCC spike-in RNA mix 1 (Ambion 4456740) was also added for a final mass of approximately 7 femtograms (fg.) per sample, a 4,000,000× dilution from stock. Samples for each source RNA were prepared in single batches. After amplification, cDNA from the entire C1 96-well plate was quantified using picogreen. C1 chips with an average yield of less than 3 nanograms were discarded. The top 15 reactor wells by cDNA concentration were selected as representative 10 pg. samples for sequencing library preparation. Another 50 wells were selected by the same criteria. These were pooled in sets of 10, generating 5 100 pg. samples for each HBR and UHR. All samples for a given source were prepared in a single sequencing library preparation batch using Nextera XT C1 protocol.

#### NuGen

HBR samples were prepared in a single batch using amplification protocol 1, generating 4 10 pg. and 4 100 pg. amplified replicates. UHR samples were prepared in two batches, using either amplification protocol 1 or 2, generating 15 10 pg. and 11 100 pg. samples (see Additional file 1). A single sequencing library preparation was performed for each batch of samples using either Lucigen NxSeq or NuGen Ovation Rapid protocol (see Additional file 1).

#### aRNA

Amplification was performed as previously described [28]. HBR samples were prepared in 4 batches from separate dilutions of reference RNA, generating 19 10 pg. and 3 100 pg. amplified replicates. ERCC spike-ins were added to 5 of the 10 pg. replicates before amplification at a dilution of 4,000,000× from stock. UHR samples were diluted and amplified in 2 batches from separate dilutions of reference RNA, generating 12 10 pg. and 7 100 pg. amplified replicates. (Additional file 1). A single sequencing library preparation was performed using Illumina TruSeq Stranded mRNA protocol modified to begin with amplified aRNA. A small numbers of reads were assigned to ERCC transcripts in replicates from the batch where ERCCs had been added that did not have spike-ins added (average of 0.5% of the number of reads assigned in spiked samples). 18 additional HBR 10 pg. replicates were amplified using aRNA for protocol optimization experiments (see Additional file 13). These samples were treated separately and were excluded from primary analysis.

#### Bulk UHR and HBR

For each reference RNA, three sequencing libraries were generated from bulk material at the same laboratory as the SmartSeq Plus replicates. Cytoplasmic and mitochondrial ribosomal RNA (rRNA) were depleted using Ribo-Zero Gold as part of Illumina TruSeq Stranded Total RNA protocol. Samples were sequenced on Illumina HiSeq 2000. We also accessed publicly available bulk sequencing of HBR and UHR generated using poly-A selected RNA generated using standard Illumina mRNA-Seq protocol and sequenced on Illumina HiSeq 2000 using 100 bp. paired-end reads. (SEQC/MAQC-III Consortium, 2014, GEO accession numbers: GSM1362002-GSM1362029 (HBR), GSM1361974-GSM1362001 (UHR), downloaded in May 2015 [25].) These samples were generated as part of a larger experiment to evaluate bulk RNA sequencing where poly-A sequencing was performed at seven sites. For each HBR and UHR, four replicate libraries generated at the NYG site were used. Sequenced read data for each source were pooled. We additionally used publicly available PrimePCR measurements generated by the SEQC/MAQC-III Consortium using UHR and HBR RNA (SEQC/MAQC-III Consortium, 2014, GEO accession number: GPL18522, downloaded in Feb. 2015 [25]) to evaluate our reference gene abundance estimates.

Because of differences in experimental design, direct comparison across methods of precision and the effect of input RNA abundance is difficult. For example, input RNA amount as a factor have different meanings for the different amplification methods: for SmartSeq Plus, because 100 pg samples were constructed by pooling 10 pg. samples after cDNA amplification, any resulting effects involve library construction, while for aRNA and NuGen resulting effects reflect both cDNA amplification steps and library steps.

### Alignment and quantification

Low confidence nucleotides (with Phred score less than 20) were treated as unknown and replaced with Ns. Unknown nucleotides (Ns) at the ends of reads were trimmed. Poly-A and method-specific adapter sequences were trimmed from the 3′ end of reads using in-house software [29]. Reads were aligned to the human reference genome, build hg19, and to ERCC spike-in transcript sequences using STAR (Spliced Transcripts Alignment to a Reference) aligner [21]. The STAR aligner was developed to map the spliced reads expected from RNA-seq to non-contiguous regions of the reference genome. In brief, STAR uses a two-stepped approach to align a spliced read to the genome. First, STAR performs a “seed” search, in which it searches for the longest substring of the read that exactly matches to the reference genome. This search is repeated for the unmapped portion of the read. Second, STAR stitches together seeds identified in the first step in a manner that allows for mismatches and that considers the genomic proximity of alignments and, optionally, splice-site annotations. We provided STAR with GENCODE18 annotations to generate a splice-junction loci database for use in alignment. We retained reads that aligned to at least 40% (paired-end) or 60% (single-end) of trimmed length or 30 bp, whichever was greater. In addition, we discarded reads with greater than 30% mismatched positions in trimmed length. Uniquely aligned reads were assigned to GENCODE18 gene annotations and to ERCC transcripts using HTSeq and htseq-counts. Reads overlapping multiple annotations were assigned to a single gene or discarded using the intersection non-empty method [22]. We normalized raw read counts for differences in sequencing depth using size-factors estimated by the method proposed by Anders and Huber and implemented in DESeq [23] after filtering genes as described in *Excluded and ambiguous genes*, below. aRNA sequenced data retained RNA strand information, but we did not use this information in quantification so that that all methods were analyzed consistently. For protocol optimization analysis (Table 2), aRNA samples were re-quantified using strand information where applicable. Each method demonstrated different dependence of read counts on gene length (Fig. 2h), so no single length normalization procedure was appropriate, hence the analyses were completed without length normalization.

To estimate input RNA abundances, raw sequencing data from all three ribosome-depleted bulk HBR or UHR replicates were pooled resulting in a single sample for each HBR and UHR with sequencing depth of ~400 million reads. Sequencing characteristics of bulk RNA sequencing are relatively well known and we used a model theoretic method to estimate reference gene expression, as implemented in RSEM (RNA-seq by Expectation-Maximization, version 1.2.18, using Bowtie version 1.1.1) strand-specific quantification [24, 30]. Poly-A tails were not added to transcripts. RSEM gene abundances were normalized to transcripts per million (TPM). 50.4% and 51.1% of reads aligned to genes for HBR and UHR, respectively.

We validated the robustness of the RSEM abundance estimates by comparing them to estimates generated using two additional algorithms. First, we used HTSeq and htseq-counts [22] in the intersection non-empty mode as described above. This method makes few assumptions about the distribution of sequencing reads along transcripts. Second, we used a modified version of Maxcounts [31], a method designed to be robust to differences in sequencing protocol and each gene was assigned the 95%ile depth of coverage value across covered exons. For both HTSeq and Maxcounts, quantification was strand-specific and estimates were normalized to reads per million (RPM). Counts were also compared to PrimePCR measurements (see *Experimental design*). To compute gene abundance estimates using PrimePCR, we removed undetectable genes (C_T_ > 35, based on a C_T_ of 35 for one DNA molecule [25]) and then subtracted 35 from each gene’s C_T_ value to generate log_2_ number of molecules, which were then converted to log_10_ units. Genes with multiple reported C_T_ measurements were removed, leaving 11,788 (UHR) and 11,572 (HBR) gene measurements for analysis. Pairwise scatter plots and correlations can be found in Additional file 3. All quantification algorithms provide similar estimates. We used RSEM quantification throughout because this method provides isoform expression level estimates, which allow more fine-tuned estimates of gene characteristics (such as GC content and length).

Ribosomal and mitochondrial RNA were depleted from bulk HBR and UHR samples (see *Experimental Design*). We compared estimated RNA abundances based on these samples to abundance based on samples generated using poly-A RNA to determine whether the method of RNA selection substantively affected abundance estimates. Expression estimates were similar across library preparation methods and the library generated with ribosomal and mitochondrial depletion demonstrated the greatest similarity with qPCR measurements (Additional file 3, panel B). RSEM expression level estimates based on ribosomal and mitochondrial RNA depleted samples were used as “truth” throughout.

### Excluded and unambiguous genes

We excluded ribosomal genes, genes with short isoforms, and genes on the mitochondrial chromosome, as described in the main text. Inferences made by bioinformatics methods may affect sensitivity, precision, and quantification accuracy for any individual gene. We identified a stringent set of genes to which reads could be uniquely aligned, in order to focus on sensitivity, precision and accuracy of the molecular measurements. Identified genes did not overlap in genomic positions with exons from any other annotated gene on either strand and could be aligned to uniquely across the entire gene. As a measure of mappability we used the GENCODE CRG Alignability track for reference genome hg19, generated by the ENCODE project and downloaded as a bigwig file from the UCSC Genome Browser on Sept. 23 2014 [32]. This track contains sliding windows of k-mers and a record of how many locations in the genome each k-mer aligns using the GEM aligner allowing up to two mismatches. We used k equal to 50 nucleotides because the minimum read length in this study was 50 base pairs. Genes where all sliding windows align to only one location were considered uniquely alignable.

### Expected number of molecules in diluted replicate

See *Results* for an overview of the approach.

To estimate the mass of RNA targeted for cDNA synthesis, we followed a previously described method [2]. For each SmartSeq Plus dilution replicate, we calculated the percent of reads assigned to ERCC transcripts, with respect to the total number of reads assigned to genes that were retained after filtering. (SmartSeq Plus samples were used because all replicates included ERCC spike-ins.) We divided the known ERCC mass (7.12 or 71.2 femtograms) by the average percentage of reads assigned to ERCC transcripts to get the total mass of targeted transcripts and ERCC molecules and therefore the mass of targeted transcripts. By this method, we estimated the following masses for targeted molecules: 0.24 pg. (HBR 10 pg. replicates), 2.4 pg. (HBR 100 pg. replicates), 0.26 pg. (UHR 10 pg. replicates) and 2.6 pg. (UHR 100 pg. replicates).

To find the expected number of molecules in a diluted replicate, we first found the weighted average transcript length for each HBR and UHR. To do this, we took the average of transcript lengths weighted by transcript relative expression levels, across all transcripts. (Both transcript lengths and relative expression levels were estimated by RSEM on bulk HBR and UHR.) We then found the expected number of molecules in a diluted replicate by dividing the mass of targeted cDNA in a replicate (found in step 1) by the mass of the average transcript. The average transcript length for HBR, based on RSEM relative gene expression level estimates, was 1,535.56 nucleotides (average transcript mass of 8.175 × 10^−7^ pg. and 288,600 molecules in 10 pg. replicate); for UHR, it was 1,348.39 nucleotides (average mass of 7.179 × 10^−7^ pg. and 364,762 molecules in a 10 pg. replicate).

Finally, to find the expected number of molecules for each gene in a diluted replicate, we multiplied the relative frequencies of gene expression (estimated by RSEM for each bulk HBR and UHR) and the expected number of molecules in a diluted replicate (found in step 2).

We repeated this analysis using five aRNA HBR 10 pg. samples that contained ERCC spike-ins to estimate the mass of targeted mRNA in a diluted HBR replicate. The mass of targeted mRNA was estimated to be 0.15 pg. (HBR 10 pg. replicates). We used RSEM relative gene expression level estimates for poly-A selected bulk HBR samples to estimate the number of targeted mRNA molecules. The average mRNA transcript length in HBR was estimated to be 1,968.73 nucleotides (average mass of 1.048 × 10^−6^ pg. and 143,631 molecules in a 10 pg. replicate).

For ERCC molecules, the expected number was calculated directly from the known mass of spiked-in materials and the known molarity of each spike-in transcript.

### Genomic distribution of sequenced reads

Genomic regions were assigned to eight categories hierarchically so that each region was assigned to only one category and so that each read was greedily categorized in the following order: rRNA exon, rRNA repeat, exon (excluding rRNA), intron, flank, intergeneic. Regions were defined based on the following annotations. Exons and introns were assigned based on GENCODE18 annotations. Flanks were assigned to 5 kilobases up- and down-stream from gene terminals. rRNA refers to GENCODE18 annotations with “rRNA” as the gene_type, which includes 5S pseudogenes. rRNA repeat refers to RepeatMasker annotations for the rRNA class of repeat. RepeatMasker annotations for reference genome hg19 were downloaded from UCSC table browser as a gtf file from the UCSC genome browser on June 23, 2015. Remaining regions were classified as intergenic. Primary alignments for all reads, including multimapping reads, were assigned to these regions using htseq-counts [33]. The STAR aligner assigns a single primary alignment to each read, with multi-mapping reads assigned the alignment with the best alignment score, if only one such alignment exists, or a randomly selected alignment from the set of best alignments. (Multi-mapping reads were included for this analysis because many rRNA regions demonstrate substantial similarity such that it was difficult to uniquely align reads to these regions.) Haplotype and random chromosomes were excluded.

### Number of detected genes

Genes not observed in the bulk (TPM = 0) were ignored, so that 28,980 genes were included in analysis for HBR replicates, and 31,263 for UHR replicates. We used the R package poibin to find a 95% CI for the expected number of genes in a diluted replicate. We performed simulations of the dilution experiment to check robustness of the result to violation of the independence assumption. Simulation results matched theoretical results (data not shown). We performed this analysis both assuming that total RNA was targeted for capture and assuming mRNA was targeted for capture (see *Expected number of molecules in diluted replicates*, above). Because UHR aRNA dilution replicates did not contain ERCC spike-ins, we could only estimate mRNA expectation for HBR. Low-depth *in silico* samples were generated for each dilution replicate by randomly subsampling 500,000 reads from the collection of all reads that were uniquely assigned to genes. (Note that this corresponds to a total read depth of roughly 10^6^ per sample).

### Gene traits

We compiled a set of gene characteristics for use in bias exploration. Traits calculated include GC content and length, both known sources of bias for bulk RNA sequencing [34]. Poly-T priming was used by aRNA and SmartSeq Plus and may introduce a bias for genes with internal stretches of adenosines, and so we also computed the presence or absence of an internal A-hexamer (6 or more sequential As). RNA secondary structure may hinder biochemical reactions and we assigned a score for the average strength of local secondary structure. To do this, we calculated the minimum free energy predicted by Vienna RNAFold (version 1.7.2) [35] for 100 nucleotide-sliding windows along the length of each isoform (step size of 1 nucleotide) and reported the average across all windows. All traits are calculated based on GENCODE18 annotated isoforms. Genes were assigned the average of isoform traits, weighted by the relative expression level of isoforms estimated by RSEM quantification of bulk HBR or UHR. We also calculated two metrics of bioinformatics complexity for each gene. As a measure of alignment complexity, we calculated the fraction of 50 base pair windows that were reported to be uniquely alignable in the GENCODE CRG Alignability track [36] (see *Excluded and unambiguous genes*, above). As a measure of quantification complexity, we calculated the fraction of the gene body that overlaps with another annotation on either strand. Both of these metrics were calculated over the union of exons for each gene.

### Detection logistic regression

For model fitting, we used computationally unambiguous genes (see *Excluded and unambiguous genes*, above) that were observed in bulk HBR or UHR. Genes within the upper or lower 2.5%ile value for any biophysical trait were excluded so that covariate ranges were well sampled. After filtering, 5,645 genes were included in analysis. The analysis was performed on 10 pg. dilution replicates. 100 pg. dilution replicates were not included because of the small sample size of these groups and because of differences between groups in how these dilution replicates were generated (see Fig. 1a and *Experimental design*). A single model was fit containing both HBR and UHR dilution replicates, in order to increase sample size and simplify analysis. A random 90% of the data were used in model development and fitting, with the remaining 10% used to assess model fit. Final sample size for model development was 323,194 observations and for validation it was 45,486 observations.

To determine the best parametric form for each independent variable we followed the multivariate fractional polynomial method. In brief, this method (developed by Royston & Altman, 1994) searches a small range of possible polynomial functions of each independent variable to identify the transform that results in the best model, defined as having the largest log-likelihood. Both one- and two-term transforms can be tested. Before selection of a “best” transform, fit models using transformed variables are compared to the linear case (and to each other, if both a one- and two-term transformation are considered) using a likelihood ratio test (here the null hypothesis of no difference in fit was rejected at *p* < 0.001). See Hosmer et al. [37] for more details. In a multivariate case, transformations are tested on individual covariates iteratively in the context of the multivariate model in order of decreasing significance, retaining selected transformations for previously tested covariates. Once all variables have been tested the process repeats, beginning with the previously identified best transforms, until no additional changes are significant. We used a closed test procedure for determining significance (see Hosmer et al.), permitting two-term transformations for the number of molecules and GC content. Single-term transforms were permitted for gene length, strength of local secondary structure and sequencing depth for the sake of model simplicity and interpretability. We used the R mfp package for this analysis [38]. For selecting parametric form, all samples were treated together, ignoring amplification method. By this method, the selected model is:

$$ \mathsf{Logit}\left(\mathsf{E}\left(\mathsf{Y}\Big|\mathsf{M},\mathsf{L},\mathsf{G},\mathsf{S},\mathsf{A},\mathsf{D}\right)\right) = {\mathsf{\beta}}_{\mathsf{0}} + \left({\mathsf{\beta}}_{\mathsf{1}}\surd \mathsf{M}\right) + \left({\mathsf{\beta}}_{\mathsf{2}}\mathsf{log}\left(\mathsf{M}\right)\surd \mathsf{M}\right) + \left({\mathsf{\beta}}_{\mathsf{3}}\mathsf{log}\left(\mathsf{L}\right)\right) + \left({\mathsf{\beta}}_{\mathsf{4}}{\mathsf{G}}^{\hbox{-} \mathsf{2}}\right) + \left({\mathsf{\beta}}_{\mathsf{5}}\mathsf{log}\left(\mathsf{G}\right)\ {\mathsf{G}}^{\hbox{-} \mathsf{2}}\right) + \left({\mathsf{\beta}}_{\mathsf{6}}\left(\mathsf{S} + \mathsf{39.1}\right)/\mathsf{10}\right) + \left({\mathsf{\beta}}_{\mathsf{7}}\mathsf{A}\right) + \left({\mathsf{\beta}}_{\mathsf{8}}{\left(\mathsf{D}/\mathsf{10}\right)}^{\hbox{-} \mathsf{2}}\right) $$

where M represents the expected number of input molecules in a diluted replicate, L represents the gene length (in kilobases), G represents the gene GC content, S represents the gene strength of local secondary structure (kcal/mol, shifted and scaled for stability), A indicates the presence of an A-hexamer within the gene body, and D represents the sequencing depth (per million reads, scaled for stability).

In the final model, amplification method was encoded as dummy variables so that method -specific coefficients were found for all independent covariates, with the exception of sequencing depth. We fit a single coefficient for depth across all methods to increase the covariate range. The final model was fit excluding 17 large influence genes (having Cook’s Distance >0.001 for at least two observations in each of at least two methods) using R built-in glm function with family (error model) set to binomial [39]. The final model can be found in Additional file 5. Model fit was assessed using normalized Chi-Square (proposed by Osius and Rojek) and normalized Sum-of-Squares goodness-of-fit statistics, evaluated on a random 10% of the data excluded from model development (Additional file 6, and see Hosmer et al. for details). To assess the benefit of including biophysical and sample covariates, in addition to the expected number of input molecules, we calculated the area under the receiver operating characteristic curve (AUC) for classification using the model, and separately for classification based on the expected number of input molecules alone. AUC provides a measure of the probability that the classifier will assign a higher score to a randomly selected detected gene than a randomly selected undetected gene. AUC average and standard deviations were calculated over 10,000 bootstrap replicates. To determine whether the model was sensitive to read length or paired end status, we calculated fit statistics for data truncated *in silico* to 50 base pair single-end reads (Additional file 6). We additionally tested extension of model to ERCC spike-in molecules (using SmartSeq Plus and aRNA 10 pg. dilution replicates containing spike-ins) and to dilution samples beginning with 100 pg. input RNA (Additional file 6). For these additional validations, a random 5,000 observations were used to calculate fit statistics. For tests of extension to 100 pg. data, SmartSeq Plus samples were excluded because these samples were not generated using 100 pg. input RNA for cDNA generation and amplification, but by pooling ten 10 pg. diluted replicates before sequencing library preparation, and so were not appropriate for the modeled process. In all cases, validation statistics were calculated based on predictions for genes within covariate ranges used in model fitting and excluding 17 identified large influence genes. For ERCC samples, this meant that four transcripts shorter than 300 nt. were excluded. Also, because the ERCC molecules span a 10^6^ range while transcriptomes at a single-cell level span ~10^3^ range, 2.5%ile trimming based on input molecules means that only 50 out 92 transcripts were used. While the expected number of input molecules is a very good predictor of gene detection, addition of the remaining independent variables improved prediction (Additional file 6). All additional independent covariates also contributed significantly to the model. The model was not sensitive to read-length or paired-end status: it fit data truncated *in silico* to 50 base-pair single-end reads well (Additional file 6). The model did not fit ERCC or 100 pg. dilution replicates well (Normalized Chi-square goodness-of-fit test *p* < 0.05); however, it still improved prediction accuracy in these cases compared to using the number of input molecules alone for prediction (Additional file 6).

When examining the effect of the number of input molecules on the probability of gene detection, the remaining covariates were set to median values (gene length of 1.05 kilobases, GC content of 0.49, average strength of local secondary structure of −24.7 kcal/mol, no internal A-hexamer, sequencing depth of 17.1 million reads). To calculate the effect of increasing sequencing depth on percent genes detected, gene detection probabilities were calculated for all genes included in regression analysis (using gene-specific covariate values) at each examined depth. The expected number of genes detected is the sum of detection probabilities over all genes. To calculate a molecular detection rate for aRNA with respect to poly-adenylated mRNA molecules, we fit a logistic model with the same functional form using expected number of input molecules calculated from bulk poly-A HBR samples, gene detection data from aRNA HBR 10 pg. dilution replicates, and fixing the depth coefficient to the value estimated in the above analysis.

### Sensitivity outliers

We calculated the squared deviance residual for each observation as a measure of fit, using the logistic model described above. The sum of squared deviance residuals is equivalent to the likelihood ratio test statistic comparing the saturated model with respect to the fitted model, and the sum of squared deviance for a subset of observations can be considered the contribution of this set of observations to overall model fit. To find method-specific problematic genes, we calculated the average squared deviance residual for each gene over all samples for each method separately. For each method, we classified genes with average squared deviance residual larger than 4 as outliers. We repeated outlier identification for computationally ambiguous genes within the range of covariates used in model fitting (*n* = 28,270).

### Coverage

Nucleotide-level coverage was calculated for each gene in the R programming environment [39] and using Bioconductor libraries GenomicRanges and Rsamtools [40–42]. Coverage was calculated based on uniquely aligned reads only. Only computationally unambiguous genes were used. Additionally, only genes with a single annotated isoform were used in this analysis.

We calculated the observed per nucleotide coverage scaled by the expected coverage as a function of absolute 3′ to 5′ position within a gene. (We chose this orientation because Smart-Seq and aRNA use poly-T priming, so that at least some cDNA priming occurs at the 3′ end of genes). HBR and UHR dilution replicates were treated together. Replicates were grouped by method and by input amount. Each gene in each sample was considered an independent replicate observation of gene coverage. Genes were filtered to include only those observations with an average of at least 2× coverage per nucleotide. Genes were aligned from the 3′ end, so that the per nucleotide sample size decreased from 3′ to 5′, resulting in increased variance in estimates from 3′ to 5′. Nucleotide positions were filtered to include only those with at least 25 replicate observations, which means that for some genes 5′ data was excluded. For each gene, per nucleotide coverage was normalized so that the expected coverage at each position was 1×. For each nucleotide position, the expected value is equal to the number of observations at that position and the observed value is the sum of normalized observed values at that position across observations. Using this this scheme, each gene of at least length *i* contributes equally to the observed coverage at position *i*, regardless of expression level. The result is positional observed/expected coverage values.

To examine patterns of gene coverage as a function of expression level, genes were grouped genes by average per nucleotide coverage. We calculated the average per nucleotide coverage for each of 100 equally sized bins from 5′ to 3′, rather than coverage as a function of absolute nucleotide position as above, in order to observe qualitative coverage patterns occurring at the same relative position along gene bodies. For each gene, bin values were normalized to sum to one so that within an expression level category all genes contribute equally. For an experimental group, positional bins were assigned the average normalized coverage across all genes, observed in any sample within the experimental group, that fell within a given expression level category.

### Precision

We calculated Pearson pairwise correlation coefficient and Kendall tau pairwise rank correlation coefficient across dilution replicates as a measure of similarity across replicates. The Pearson correlation coefficient is sensitive to large-valued outliers, while the Kendall correlation coefficient is robust. In brief, Kendall correlation is calculated as follows. For each pair of genes the pair is categorized as concordant if the relative ranks of the gene pair are the same for both samples and discordant otherwise. The coefficient reports the fraction of all pairs that are concordant less the fraction that are discordant. For both correlation coefficients, zeros were treated as missing values, such that only genes observed in both members of a pair were included in the calculation. Correlations were calculated on depth-normalized read counts (as described in *Alignment and quantification*, above).

To characterize measurement precision, we performed least-squares regression of the empirical standard deviation on the empirical mean. We used computationally unambiguous genes to fit these models. Additionally, we included only genes with >95% probability of presence in a diluted replicate, excluded gene detection outliers and trimmed the upper and lower 2.5%ile by mean value for model fitting. Both the average and standard deviation were log-transformed for normality of residuals. After all filtering, at least 1,100 genes were used to fit the model: log_10_(standard deviation) = a + b * log_10_(mean). Sample sizes ranged from 1,149 to 1,269 genes. A separate model was fit for each experimental group. Because 100 pg. experimental groups have small sample sizes (for most, *n* < =5) and so provide unstable estimates of variance due to missing values, we performed this analysis on 10 pg. groups only. The NuGen HBR 10 pg. sample size is also quite small (*n* = 4) and was excluded.

To characterize biases in experimental variation we selected a subset of genes where empirical standard deviation was not well predicted by the mean. Genes with residuals in the upper or lower 5%ile were categorized as having high or low experimental variability, respectively. Remaining genes were used as background for enrichment tests for biophysical characteristics. For enrichment tests of GC-content, length, and secondary structure, we calculated the Hodges-Lehmann estimate of difference in location to provide an estimate of the magnitude of in location between test and background gene. This metric estimates the median difference between the two groups.

For each method, genes with residuals within the upper or lower 1%ile were classified as outlier genes with unexpectedly high or low experimental variability. Outliers were identified for each experimental group, and then merged across input amounts for each RNA source by taking the union of identified outliers. We considered both computationally unambiguous genes and also computationally ambiguous genes, excluding those computationally ambiguous genes with mean expression outside the range used in model fitting.

Principal components analysis was performed on sample covariance matrix calculated using zero-corrected log-transformed read counts for computationally unambiguous genes with non-zero counts in at least on sample and using the R prcomp function. Each PCA included the appropriate bulk HBR or UHR. RSEM-estimated relative frequencies were normalized to the same scale as the diluted replicates using the DESeq method for estimating size factors, as described above. Bivariate normal 95% confidence ellipses were calculated for each experimental group using the R dataEllipse function from the car package [43].

### Accuracy

Sample sizes (number of genes) for analysis in Fig. 4d-n, given filtering described in plot legend, were the following: HBR: *n* = 1,339 (10 pg.) and 2,797 (100 pg.); UHR: *n* = 1,243 (10 pg.) and 2,614 (100 pg.) As stated, in evaluation of gene measurements in individual dilution measurements genes with zero read counts were excluded. For evaluation of average gene measurements, zero values in individual replicates were retained. RSEM-estimated relative frequencies were treated as true relative expression values for each gene. These were normalized to the same scale as the diluted replicates using the DESeq method for estimating size factors, as described above. Wide boxes in boxplots of fold deviation in Fig. 4d–f include values for all samples in an experimental group.

To identify method-specific biases in accuracy, we calculated the median fold deviation for each gene across dilution replicates within each experimental group. Genes with fewer than three observations were removed. Of the remaining genes, those with median fold deviation in the upper or lower 5%ile were categorized as overestimated and underestimated, respectively. Remaining genes were used as background for enrichment tests for enrichment. For each method, genes within the upper or lower 1%ile were classified as outlier genes with poor accuracy. Outliers were identified for each experimental group, and then merged across input amounts for each RNA source by taking the union of identified outliers. We repeated outlier identification using computationally ambiguous genes, following the same filtering criteria described above.

To generate density scatter plots of gene read counts in individual dilution replicates, measurements from all 10 pg. dilution replicates for a given method were pooled. The density scatter plots were generated using the R densCols and KernSmooth::bkde2D functions. These functions estimate local density using a binned approximation to a 2 dimensional kernel density with a bivariate Gaussian kernel. log_10_ read counts were used. Low-depth *in silico* samples used in Additional file 11 were generated as described above (“*Number of detected genes*”). For density scatter plots of average read counts, averages were taken separately for HBR and UHR 10 pg. dilution replicates. Averages for HBR and UHR were pooled before density calculation.

### Protocol variations

To evaluate the effect of removing purification of initial cDNA, 12 additional HBR 10 pg. dilution replicates were generated. 6 were generated using the same cDNA protocol as the primary aRNA samples, in which initial cDNA is purified using a MinElute column. 6 were generated without this purification step, with adjusted molarity for aRNA amplification to accommodate the change in reaction volume. Each set of 6 included 3 replicates generated using 13 rounds of PCR amplification during sequencing library preparation and 3 using 15. In this analysis, differences in PCR treatment were ignored.

To evaluate the effect of reducing rounds of cDNA amplification, 5 additional HBR 10 pg. dilution replicates were generated using 2 rounds of IVT amplification (rather than 3). All amplified material was used as input for sequencing library preparation. Additionally, these samples were generated without initial cDNA purification and using 15 rounds of PCR during sequencing library preparation (rather than 13). These data were compared to 3 replicates generated using 3 rounds of aRNA amplification, and otherwise following the same protocol. To evaluate an optimized aRNA protocol, excluding initial cDNA purification and reducing rounds of amplification, the same 5 HBR 10 pg. dilution replicates used to examine the effect of reducing rounds of IVT amplification were compared to the primary HBR 10 pg. aRNA data.

To examine the effect of ERCC addition, 10 replicates beginning with 10 pg. HBR total RNA were amplified using aRNA. In 5, ERCC spike-in controls were added with reference RNA at a final dilution of 1:4,000,000. Samples generated in ERCC optimization showed evidence of cross-contamination, with counts assigned to ERCC transcripts (total ERCC counts: 892–1,457) at appropriate relative abundances for samples generated without addition of ERCC controls.

The effect of strand-specific sequencing was evaluated by re-quantifying aRNA HBR 10 pg. samples using strand information.

## Additional files

Additional file 1: Control dataset sample identification, protocol information, and RNA sequencing stats. Experimental group, protocol information and RNA sequencing statistics for each sample used in primary analyses. Alignment statistics were based on STAR alignment to hg19 and were with respect to reads retained after trimming for primer or poly-A sequences [21]. (XLS 62 kb)
Additional file 2: Computationally unambiguous genes. Genes to which reads can be uniquely assigned. See the *Excluded and unambiguous genes* section in *Methods* for details on classification. (PDF 516 kb)
Additional file 3: Accuracy and robustness of estimated reference HBR and UHR RNA expression levels. A. Consistency of abundance estimates by three quantification algorithms relative to publicly available PrimePCR measurements (see *Methods*). Scatters show log_10_ reads per million (HTSeq [22] and Maxcounts [31]), log_10_ transcripts per million (RSEM), or log_10_ molecules (PrimePCR). Upper quadrants indicate Pearson correlation (R) of log-transformed estimates. Pairwise zeros were treated as missing values. Estimates were based on combined raw reads from 3 bulk reference samples generated using ribosomal depletion for each HBR and UHR. RSEM estimates were used as reference throughout. B. Accuracy and robustness of expression estimates across library preparation methods: ribosomal-depletion (combined *n* = 3 samples per HBR and UHR) and poly-A RNA selection (combined *n* = 4 samples per source). See *Methods* for sample information. Scatters as in A using RSEM expression level estimates for each library preparation method. Ribosomal-depletion samples were used as reference throughout. Abbreviations: Human Brain Reference (HBR), Universal Human Reference RNA (UHR). (PDF 469 kb)
Additional file 4: Number of detected genes for low-depth *in silico* samples. As Fig. 2a, except that each dilution replicate has been subsampled to a depth of 500,000 unique genic reads. (XLS 74 kb)
Additional file 5: Gene detection logistic regression model. See model details in *Methods*. *Abbreviations*: M expected number of input molecules; L gene length (kilobases); G gene GC content; S strength of gene local secondary structure (kilocalories per mole); hasA presence of A-hexamer internal to gene body; D Depth (per 10,000,000 reads); *S.E.* standard error; *Wald Z* Wald test statistic; *Pr(>|Z|)* Wald test *p*-value. (XLS 40 kb)
Additional file 6: Gene detection logistic regression fit and validation. Model was fit using randomly selected 90% of 10 pg. data, excluding 17 large influence genes. Fit was evaluated on the remaining 10% of the data. Fit was also evaluated on sequence data that was *in silico* truncated to 50 base pair single end (“Truncated”), ERCC read counts (“ERCC”), and 100 pg. dilution replicates (“100 pg.”). AUC (area under receiver operating characteristic curve) reported as mean values ± 2 Sd. calculated over 10,000 bootstrap samples. AUC (molecules) predicts detection based on number of input molecules alone. See *Methods* for further details. (XLS 29 kb)
Additional file 7: Probability of gene detection. Based on model described in *Methods*. Remaining covariates set to median value. (PDF 34 kb)
Additional file 8: Gene detection outliers. Genes that are problematic for detection. See *Methods* for classification of outliers. “Gene set” indicates whether gene is classified as computationally unambiguous (1) or not (2). “Detected/undetected” indicates whether the gene is unexpectedly observed (D) or unexpectedly unobserved (U). (PDF 181 kb)
Additional file 9: Precision outliers. Genes with residuals within the upper or lower 1%ile with respect to regression of standard deviation on the mean (see *Methods*). “Gene set” indicates whether gene is classified as computationally unambiguous (1) or not (2). Only genes whose mean is within the range of fitted model were included. Column values indicate whether indicate whether the gene standard deviation is unexpectedly low (L) or high (H), given mean. (XLS 114 kb)
Additional file 10: Accuracy outliers. Genes were identified as accuracy outliers if its median fold deviation, taken across dilution replicates, was contained in the upper or lower 1%ile of all considered genes (see *Methods*). Columns labeled by single-cell protocol contain an “H” if a gene was identified as an overestimated outlier, and an “L” if a gene was identified as an underestimated outlier. “Gene set” indicates whether gene is classified as computationally unambiguous (1) or not (2). (XLS 218 kb)
Additional file 11: Measurement reliability at low sequencing depth. As Fig. 4k–m, but using low-depth *in silico* samples in place of individual 10 pg. replicates. (A) aRNA. (B) SmartSeq Plus. (C) NuGen. (XLS 329 kb)
Additional file 12: Normalized read counts and expectation for ERCC transcripts. A–B. ERCC transcripts are found along the x-axis, ordered by expected number of input molecules. Axis labels are in the format of “ERCC spike-in ID, expected number of input molecules”. Points indicate the normalized read count for one transcript in one sample. Horizontal gray lines and background gray boxes indicate the expected normalized read count and a 95% CI under a Poisson model of dilution. Wide red horizontal lines indicate mean normalized read counts across all ERCC transcripts with a common expected number of input molecule, and red boxes indicate mean ± 2 × s.e.m. (A) aRNA. (B) SmartSeq Plus. (XLS 964 kb)
Additional file 13: Optimization dataset sample identification, protocol information, and RNA sequencing stats. As Additional file 1 for samples used in protocol optimization analyses. (XLS 37 kb)
