# Supplementary figures and images for: Assessing characteristics of RNA amplification methods for single cell RNA sequencing
Source: BMC Genomics. 2016 Nov 24;17:966. doi: 10.1186/s12864-016-3300-3 (PMC5122016; doi:10.1186/s12864-016-3300-3)

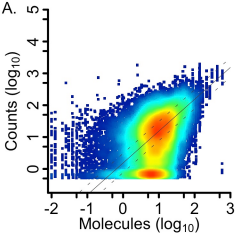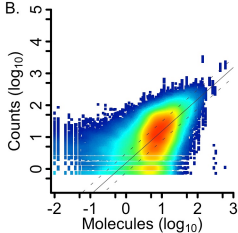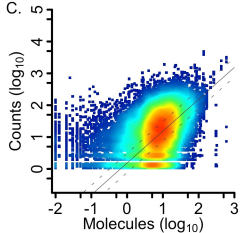

Supplement: Additional file 2: — Computationally unambiguous genes. Genes to which reads can be uniquely assigned. See the Excluded and unambiguous genes section in Methods for details on classification. (PDF 516 kb) [file 12864_2016_3300_MOESM11_ESM.pdf]

B.

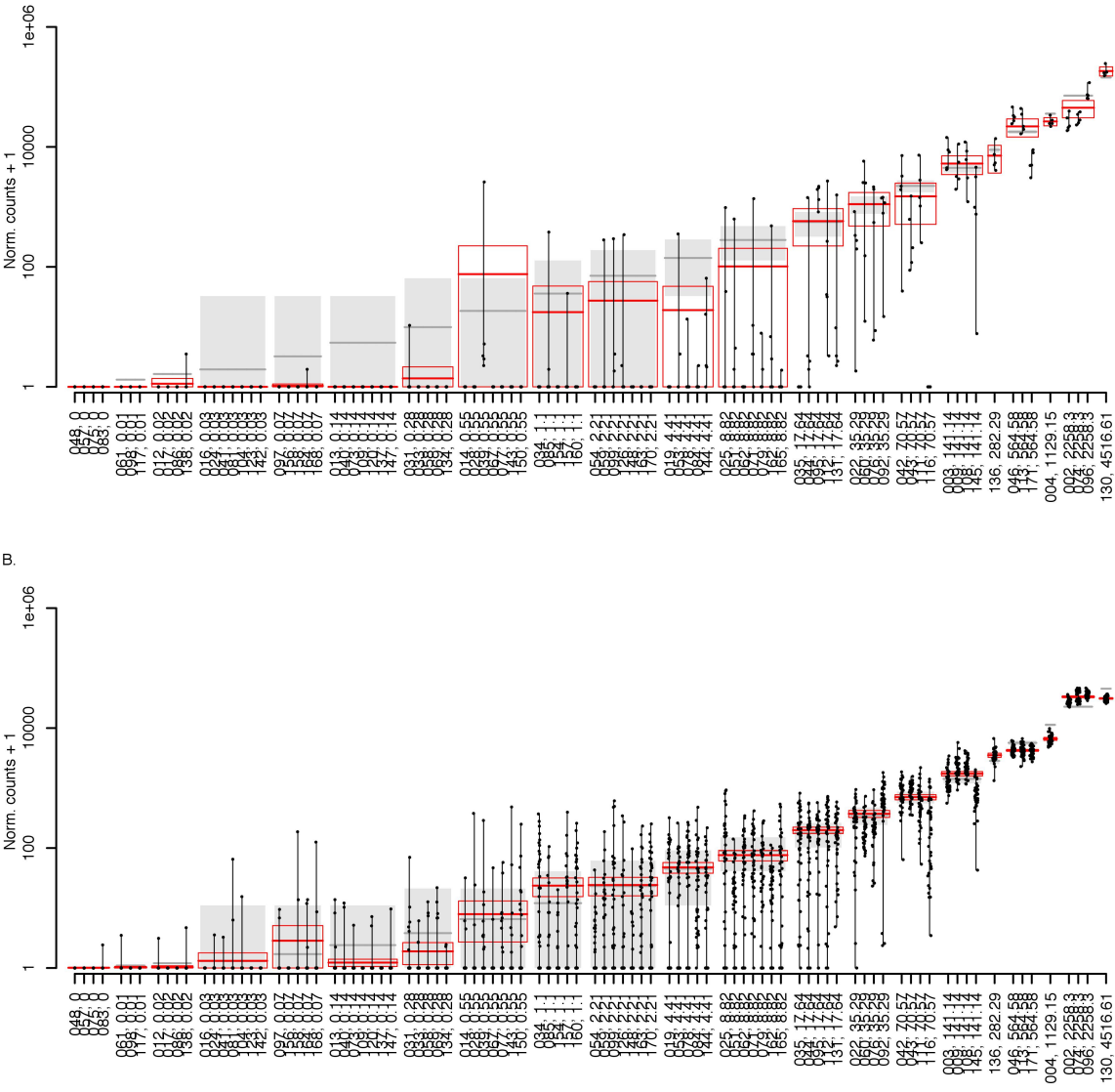

Supplement: Additional file 3: — Accuracy and robustness of estimated reference HBR and UHR RNA expression levels. A. Consistency of abundance estimates by three quantification algorithms relative to publicly available PrimePCR measurements (see Methods). Scatters show log10 reads per million (HTSeq [22] and Maxcounts [31]), log10 transcripts per million (RSEM), or log10 molecules (PrimePCR). Upper quadrants indicate Pearson correlation (R) of log-transformed estimates. Pairwise zeros were treated as missing values. Estimates were based on combined raw reads from 3 bulk reference samples generated using ribosomal depletion for each HBR and UHR. RSEM estimates were used as reference throughout. B. Accuracy and robustness of expression estimates across library preparation methods: ribosomal-depletion (combined n = 3 samples per HBR and UHR) and poly-A RNA selection (combined n = 4 samples per source). See Methods for sample information. Scatters as in A using RSEM expression level estimates for each library preparation method. Ribosomal-depletion samples were used as reference throughout. Abbreviations: Human Brain Reference (HBR), Universal Human Reference RNA (UHR). (PDF 469 kb) [file 12864_2016_3300_MOESM12_ESM.pdf]

A.

HBR

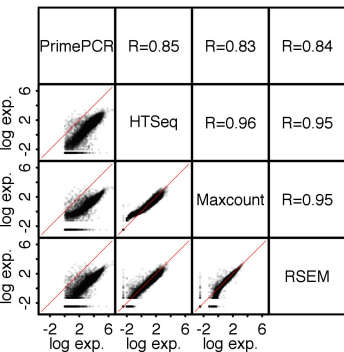

UHR

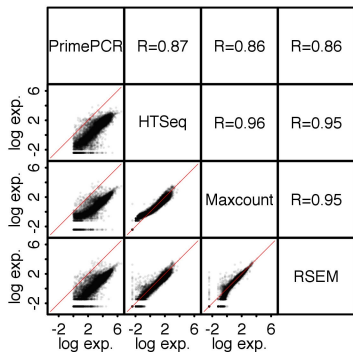

B.

HBR

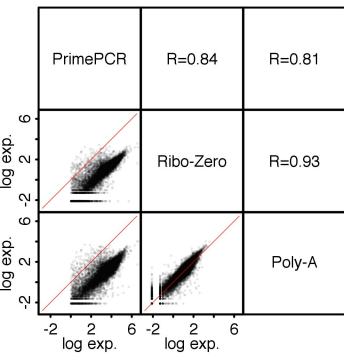

UHR

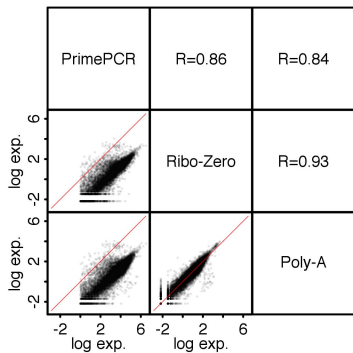

Supplement: Additional file 7: — Probability of gene detection. Based on model described in Methods. Remaining covariates set to median value. (PDF 34 kb) [file 12864_2016_3300_MOESM3_ESM.pdf]

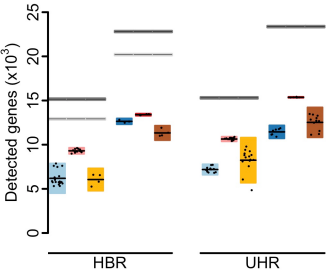

Supplement: Additional file 8: — Gene detection outliers. Genes that are problematic for detection. See Methods for classification of outliers. “Gene set” indicates whether gene is classified as computationally unambiguous (1) or not (2). “Detected/undetected” indicates whether the gene is unexpectedly observed (D) or unexpectedly unobserved (U). (PDF 181 kb) [file 12864_2016_3300_MOESM4_ESM.pdf]
